# Optimization of a Mass Trapping Method against the Striped Cucumber Beetle *Acalymma vittatum* in Organic Cucurbit Fields

**DOI:** 10.3390/insects13050465

**Published:** 2022-05-17

**Authors:** Jessee Tinslay, Marc Fournier, Isabelle Couture, Pierre J. Lafontaine, Maxime Lefebvre, Eric Lucas

**Affiliations:** 1 Biocontrol Laboratory, Biological Sciences Faculty, University of Quebec at Montreal, 141 President-Kennedy Street, Montreal, QC H2X 3Y5, Canada; fournier.marc@uqam.ca (M.F.); lucas.eric@uqam.ca (E.L.); 2 Quebec Ministry of Agriculture, Fisheries and Food, 1355 Johnson O. Street, Saint-Hyacinthe, QC J2S 8W7, Canada; isabelle.couture@mapaq.gouv.qc.ca; 3 Carrefour Industriel et Expérimental de Lanaudière, 801 Rang Bas L’Assomption Nord, L’Assomption, QC J5W 2H1, Canada; p.lafontaine@ciel-cvp.ca; 4 Research and Development Institute for the Agri-Environment, 335 Rang Des Vingt-Cinq E., Saint-Bruno-de-Montarville, QC J3V 0G7, Canada; maxime.lefebvre@irda.qc.ca

**Keywords:** integrated pest management (IPM), flower volatiles, plant semiochemicals, attractants, organic crop production

## Abstract

**Simple Summary:**

Striped cucumber beetles are the main pest of cucurbits in North America. Organic cucurbit producers face a great challenge since few effective organic-approved striped cucumber beetle control methods exist. In this study, we evaluated and improved a mass trapping method using perforated yellow jugs with commercially available odorant baits to attract striped cucumber beetles. Our goal was to maximize striped cucumber beetle captures while minimizing unwanted captures of beneficial insects. We found that baited traps attracted more striped cucumber beetles than unbaited traps, and that traps with smaller holes effectively captured striped cucumber beetles while limiting unwanted captures. Finally, we also determined an optimal bait type that should preferentially be used to capture striped cucumber beetles.

**Abstract:**

The striped cucumber beetle (SCB) *Acalymma vittatum* (F.) (Coleptera: Chrysomelidae) is a prime problem in North American cucurbit crops. While certain chemical pesticides efficiently control SCB in conventional cucurbit fields, alternative solutions are required due to the ever-evolving regulations on pesticides. For organic producers, very few control methods exist. A novel mass trapping method demonstrates the potential of controlling SCBs using floral-based semiochemical baited traps in cucurbit crops. The goals of this study were to (1) determine whether baited traps capture more SCBs than unbaited ones, and (2) optimize the trapping method by comparing different trap types and different commercially available attractants to maximize SCB captures while minimizing non-target species captures. The results of a first experiment showed that baited traps captured significantly more SCBs than unbaited ones. Baited traps also captured significantly more bees and hoverflies than unbaited ones. In a second experiment these unwanted captures were drastically reduced by using traps with ten 4 mm in diameter holes per side. Finally, a third experiment demonstrated that the attractant 40CT313 was the most efficient at capturing SCB compared to other tested lures. Overall, the optimized mass trapping technique demonstrated a potential to effectively control SCB populations in organic cucurbit crops.

## 1. Introduction

In North America, the striped cucumber beetle (SCB) *Acalymma vittatum* (F.) (Coleoptera: Chrysomelidae) is a main pest in cucurbit fields since the beginning of the 20th century [1,2]. Striped cucumber beetles have one generation per year in Canada, but their voltinism can vary according to geographical regions, reaching up to three generations per year in southern parts of the United States [3]. They emerge from their hibernation when temperatures reach 10 °C. The exact hibernation sites are still unclear; however, SCB have been found to hibernate under leaf litter and dense weeds near cucurbit fields, underground below frost level, or along river bottoms and dense weeds near river bottoms [4,5,6,7].

In spring, SCBs feed on alternative hosts, such as rosaceas, asteraceas, and legumes [3,4]. They will invade cucurbits as soon as seedlings are transplanted into fields. In Quebec, Canada, two SCB population peaks are notable during the cucurbit growth season: one in spring, when cucurbit seedlings are being transplanted to fields and hibernating adults leave intermediate host plants to aggregate onto cucurbits, and one at the end of July, which corresponds to the new adult generation that will hibernate in fall. Intervention thresholds range between 0.5 SCB per plant for cucumber (*Cucumis sativus* (L.)), kabocha and buttercup squash, which are two varieties of the species *C. maxima* (Duchesne), and spaghetti squash, *Cucurbita pepo* (L.). As for the other cucurbits, the economic threshold corresponds to one SCB per plant [8].

Striped cucumber beetles directly damage cucurbit roots, plants, and fruits through feeding and may transmit several diseases [4,9]. Bacterial wilt, caused by the bacteria *Erwinia tracheiphila*, is one of the most serious diseases transmitted by SCBs and can lead to 80% losses in cucurbit crop productions [9,10]. For Canadian organic cucurbit producers, limiting damage caused by SCBs is a difficult task since few effective options exist to control SCBs in their crops. Only two organic insecticides are listed in Organic Materials Review Institue (OMRI) of Canada for use against SCBs; the Safer’s^®^ Insecticide Trounce Concentrate and the Surround^®^ WP [11,12]. However, both insecticides present limitations. Safer’s^®^ Insecticide Trounce is a pyrethrin-based pesticide and is highly toxic to bees, which must be applied in the evening to mitigate effects on bees. Surround^®^ WP is a kaolin clay that perturbates oviposition of non-target insects and washes off during rain showers [11,13,14]. Insect-proof nets, crop rotation, trap crops, and plastic tunnels constitute integrated pest management alternatives to protect small cucurbit cultivars in Quebec (Canada), but have limitations such as the duration of use, the cost and effort required for installation [4,13,15,16,17,18].

A mass trapping method developed by Jaime Piñero and Rusty W. Lee in 2016 provides a potential alternative for organic cucurbit producers [19]. In Piñero and Lee’s study, traps baited with plant-based semiochemical lures reduced populations within conventional cucurbit fields under the economic threshold [19]. The study also showed that the trapping method captured few non-target insects, such as pollinators, which are valued allies in entomophilous crops like cucurbits [20,21,22,23]. This trapping method was used in combination with conventional pesticide control.

Insects, including SCBs, forage using both olfactory and visual cues. Plant volatiles and color may encourage mutualistic interactions, for example, pollination, or to repel antagonistic interactions, such as herbivory [24,25,26]. Consequently, plant volatiles which attract pests possess interesting potential for trapping methods [19,27,28]. For cucurbits, three volatile semiochemicals are responsible for attraction of both pollinators and SCBs: 1,2,4-trimethoxybenzene, indole, and (E)-cinnamaldehyde, also called TIC [29,30,31]. For SCB control, these volatile semiochemicals are synthesized and converted into commercially available attractants and easy to use [32,33,34]. As for visual cues, it has been previously established that yellow was the most attractive color for SCB, and traps of this color maximized captures [35].

Trap types, such as shape and color of traps, and baits, such as semiochemical lures, food and pheromonal lures, can largely influence both pest captures and bycatch of non-target species [35,36,37]. It is therefore primordial to use traps that will target pests while minimizing bycatch of other species, as traps can detriment local non-target insect populations, such as beneficial predators and pollinators [38].

The first objective of our study was to assess the potential of the semiochemical-baited traps to control SCBs in Canadian organic agroecosystems. We predicted that baited traps would capture significantly more SCBs than unbaited traps in organic cucurbit crops. The second objective was to select the optimal trap within five trap types with different number and width of openings. Our predictions were (1) that smaller openings would prevent capturing larger beneficial insects, such as bees or ladybird beetles, while still enabling SCBs to be trapped; or (2) that fewer openings would reduce the amount of SCBs captured. The final objective was to compare the efficiency of the different semiochemical-based lures commercially available, our prediction being that one commercially available attractant would attract significantly more SCBs than the other attractants tested.

## 2. Materials and Methods

### 2.1. Study Sites and Experimental Plots

The first experiment, consisting of comparing baited versus unbaited traps, was conducted during summer 2018 (26 June 2018 to 28 August 2018) in *La Bourrasque* organic farm (site 1) in Saint-Nazaire-d’Acton (Quebec, Canada) (45°74′ N, 72°65′ W). Fields were plowed the previous fall. Two plots, a cucumber (*Cucumis sativus*) field of 75 m × 5 rows and a zucchini (*Cucurbita pepo*) field of 75 m × 5 rows were tested. The rows were prepared with raised beds 1.2 m wide × 0.15 m high and covered by plastic mulch in the cucumber field. Fields were irrigated when necessary, and fertilizers were applied based on crop recommendation. Neither fungicides nor insecticides were used.

Two other experiments, one consisting of comparing trap types and the other attractant types, were conducted during summer 2019 (11 June 2019 to 29 September 2019) in *La Bourrasque* farm (Site 1) and *Samson & fils* organic farm (site 2) in Farnham (Quebec, Canada) (45°25′ N, 72°96′ W). In both sites, *Cucurbita moschata* (Duchesne; butternut squash) and *C. pepo* (delicata, spaghetti and acorn squashes, pumpkins, and zucchinis) were mixed in the rows. In *La Bourrasque* farm *Cucurbita maxima* (buttercup squash) and *Luffa* sp. were also mixed in the rows. Fields were plowed the previous fall. The rows were prepared with raised beds 1.2 m wide × 0.15 m high. In *Samson & fils* farm, there were 10 rows measuring 492 m in length, the beds were covered with plastic mulch, and areas between rows were weeded either by hand or with a row cleaner. In *La Bourrasque* farm, there were 15 rows measuring 150 m in length, no plastic mulch was used, and the beds were occasionally weeded by hand. In both sites, the native vegetation growing near the traps was mowed to ensure visibility. In both sites, plots were irrigated when required, and fertilizers were applied based on crop recommendation. In *Samson & fils* farm, the copper fungicide Cueva^®^ Commercial was applied twice, and the organic pesticide Trounce^®^ was applied once on 11 July 2019, to reduce SCB populations in the crops. In *La Bourrasque* farm, neither insecticides nor fungicides were used.

### 2.2. Pest Control Potential of the Mass Trapping Method

In this experiment, we determined whether the mass trapping method was promising for organic cucurbit productions in Quebec through a split-plot experimental design. Traps were modified from Piñero & Lee’s trap design [19]. Plastic milk jugs (3.8 L) were pierced using a 6 mm in diameter soldering iron. Holes made using this technique measured 5.94 ± 0.13 mm. Two series of ten holes were perforated horizontally on each side of the jugs. The traps were spray-painted yellow with a high gloss paint (Krylon^®^ sun yellow) to improve SCB attraction. Both zucchini and cucumber plots were split in two, paired treatments; a side protected by 6 baited traps, and a side protected by 6 unbaited traps. The lure used was Trécé TRE8276 (1,2,4-trimethoxybenzene [500 mg], indole [500 mg], and (E)-cinnamaldehyde [500 mg]; Trécé^®^, Adair, OK, USA). Each trap was filled with 1 L of unscented soapy water to drown the insects. A lure was inserted through the opening of the jug and suspended using a twist tie. The string was secured using the screw-top lid. The two treatments were distanced 15 m within a plot and traps within a treatment were installed 0.45 m above the ground and placed 5 m from one another. The traps were installed on 26 June 2018, along the edge of the ditch or forest bordering the plots. Every week, for 9 weeks, the SCBs captured in each trap were counted and the soapy water within the traps was changed. Starting on 10 July 2018, captured non-target insects, i.e., bees, hoverflies, and ladybirds, were identified and counted in situ. For each experiment, the numbers of insects captured in the treatments were compared separately for SCB, bees, hoverflies, and ladybirds. The data was non-parametric and one-way ANOVAs were used for statistical analysis.

All statistical analyses in this study were performed using R statistical software version 4.1.0 using [AICcmodavg] and [multcompView] libraries (R Core Team, 2021). Data did not require transformation

### 2.3. Selection of the Optimal Trap (Number and Diameter of Trap Entrances)

In this experiment, we compared five trap types to maximize SCB captures and to limit non-target insect captures. The traps were made following the same protocol as in the first experiment, except that the size and number of holes varied from one type to the other. The five treatments corresponding to different trap types were

1. T20-4mm: traps with two series of ten holes of 4 mm in Ø (diameter) per side;
2. T10-4mm: traps with two series of five holes of 4 mm in Ø per side;
3. T20-5mm: traps with two series of ten holes of 5 mm in Ø per side;
4. T10-5mm: traps with two series of five holes of 5 mm in Ø per side;
5. T20-6mm: traps with two series of ten holes of 6 mm in Ø per side.

The traps were installed on 18 June 2019, at both sites (site 1 and 2) and arranged in a randomized complete block design (RCBD) repeated four times. The traps were placed 5 m from one another along the edge of the ditch or forest bordering the plots and installed 0.45 m above the ground. Every week, the insects captured in each trap were collected in small alcohol-filled vials and brought back to the laboratory to be identified and counted. The soapy water within the traps was also changed weekly.

The identified captured insects were categorized into three functional groups: pollinators, natural enemies, and pests. For each experiment, the numbers of insects captured in the treatments were compared separately for SCB, bees, hoverflies, and ladybirds. The data was non-parametric, and a one-way ANOVA was used for statistical analysis. Whenever appropriate, means were separated by a post hoc Tukey’s significant differences at the *p* = 0.05 level.

### 2.4. Selection of the Optimal Lure

The attractiveness of four commercial floral-based semiochemical lures to SCB adults and non-target insects was compared in a RCBD repeated four times. The trap used for this experiment was the “T20-6mm” trap from the previous experiment. The five treatments corresponding to lure types were

1. Alpha Scents SCB lure (Alpha Scents Inc., Canby, OR, USA) composed of Indole, (E)-cinnamaldehyde and 1,2,4-trimethoxybenzene, i.e., TIC mixture [26];
2. 40CT313 5-compound lure (Distribution Solida Inc., Saint-Ferréol-les-Neiges, QC, Canada), the identities of the five compounds of the 40CT313 lure were not disclosed. This attractant is the equivalent of the AgBio 5-compound lure P313-B5 (AgBio Inc., Westminster, CO, USA), which Piñero had found to be most attractive to SCB in his research;
3. KLP lure composed of 4-methoxy-cinnamaldehyde and indole (Csalomon^®^, Budapest, Hungary);
4. Trécé TRE8276 (1,2,4-trimethoxybenzene [500 mg], indole [500 mg], and (E)-cinnamaldehyde [500 mg], i.e., TIC mixture (Trécé^®^, Adair, OK, USA);
5. No lure.

The experimental design, sampling, and statistical analysis were the same as in the previous experiment.

## 3. Results

### 3.1. Pest Control Potential of the Mass Trapping Method

**Figure 1:**
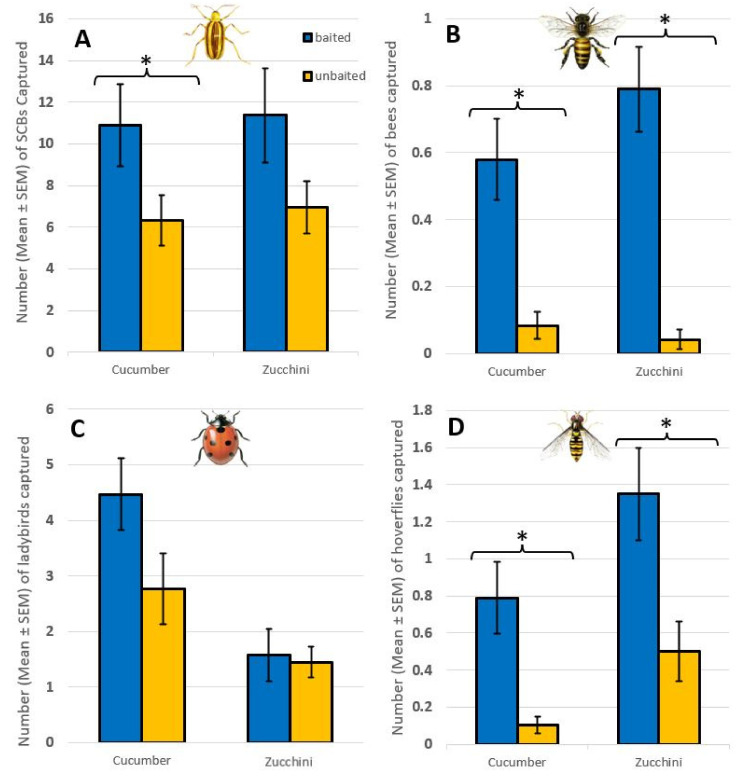
Number of SCBs, ladybirds, bees, and hoverflies captured in baited and unbaited traps in cucumber and zucchini plots (ANOVA). (**A**) SCB—cucumber: F_1,106_ = 3.94; *p* < 0.05; zucchini: F_1,106_ = 2.89; *p* = 0.09, (**B**) bees—cucumber: F_1,94_ = 15.11; *p* < 0.001; zucchini: F_1,94_ = 33.54; *p* < 0.001, (**C**) ladybirds—cucumber: F_1,94_ = 3.56; *p* = 0.06; zucchini: F_1,94_ = 0.05; *p* = 0.82, (**D**) hoverflies—cucumber: F_1,94_ = 11.80; *p* < 0.001; zucchini: F_1,94_ = 8.36; *p* < 0.01). Asterisks above bars indicate significant differences according to ANOVA tests at *p* = 0.05.

Over the eight weeks of the experiment, a total 1919 SCBs were captured in both treatments. Baited traps captured 1.70 times more SCBs than unbaited traps. The baited traps captured significantly more SCBs than unbaited traps in cucumber plots, but not in zucchini plots (ANOVA, cucumber: F_1,106_ = 3.94, *p* < 0.05; zucchini: F_1,106_ = 2.89, *p* = 0.09; Figure 1).

A total of 72 bees (Hymenoptera: Apoidea (Latreille)) were captured in both treatments. Baited traps captured 3.57 times more bees than unbaited traps, the difference between baited and unbaited traps being significant in both cucumber and zucchini plots (ANOVA, cucumber: F_1,94_ = 15.11, *p* < 0.001; zucchini: F_1,94_ = 33.54, *p* < 0.001; Figure 1).

A total of 494 ladybirds (Coleoptera: Coccinellidae; 417 *Harmonia axyridis* (Pallas), 46 *Coleomegilla maculata* (De Geer), 10 *Coccinella septempunctata* L., 12 *Propylea quatordecimpunctata* (L.), and 9 *Hippodamia* sp. (Chevrolat)) were captured. Baited traps captured 1.20 times more ladybirds than unbaited traps; however, this difference was not significant in either crop (ANOVA, cucumber: F_1,94_ = 3.56, *p* = 0.06; zucchini: F_1,94_ = 0.05, *p* = 0.82; Figure 1).

A total of 132 hoverflies (Diptera: Syrphidae) were captured in both treatments. Baited traps captured 2.16 times more hoverflies than unbaited traps, the difference between baited and unbaited traps being significant in both cucumber and zucchini plots (ANOVA, cucumber: F_1,94_ = 11.80, *p* < 0.001; zucchini: F_1,94_ = 8.36, *p* < 0.01; Figure 1).

### 3.2. Selection of the Optimal Trap (Number and Ø of Trap Entrances)

**Figure 2:**
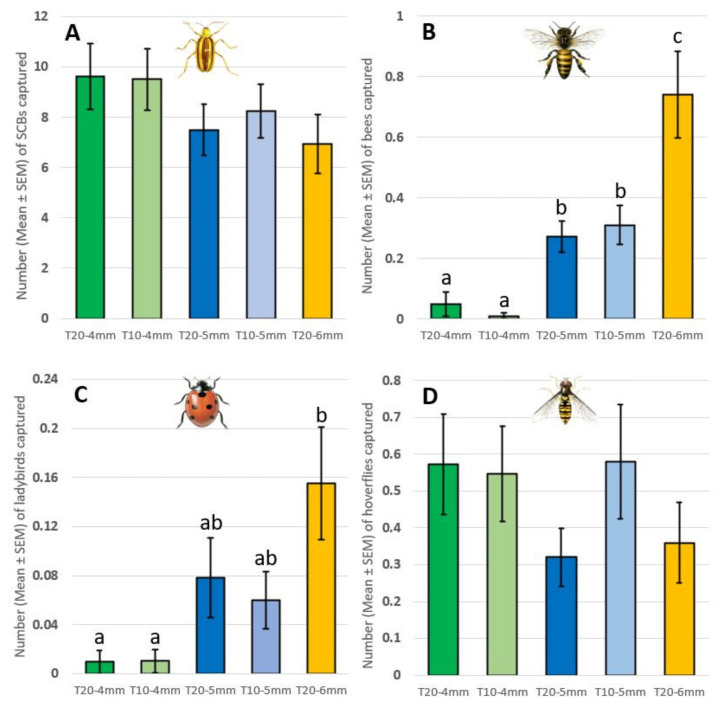
Number of SCBs, ladybirds, bees, and hoverflies captured in different trap types (ANOVA). (**A**) SCB—F_5,499_ = 0.85, *p* = 0.49; (**B**) bees—F_4,503_ = 13.78, *p* < 0.001; (**C**) ladybirds—F_4,500_ = 4.31, *p* < 0.01; (**D**) hoverflies—F_4,501_ = 0.96, *p* = 0.43). Different letters above bars indicate significant differences according to the Tukey HSD test (*p* < 0.05).

A total of 4172 SCBs were captured in this experiment. There were no significant differences in SCB captures among the different treatments (traps with two series of ten holes of 4 mm in Ø per side, traps with two series of five holes of 4 mm in Ø per side, traps with two series of ten holes of 5 mm in Ø per side, traps with two series of five holes of 5 mm in Ø per side, and traps with two series of ten holes of 6 mm in Ø per side; ANOVA F_4,499_ = 0.86, *p* = 0.49; Figure 2). Spatial blocks were a significant factor (ANOVA, F_4,499_ = 4.05, *p* < 0.05).

A total of 146 bees (107 *Apis mellifera* L., 6 *Bombus* sp. (Latreille) and 33 *Peponapis pruinosa* (Say)), were captured. Traps with 6 mm entrances captured significantly more bees than traps with 4 mm and 5 mm entrances. Traps with 5 mm entrances captured significantly more bees than traps with 4 mm entrances (ANOVA F_4,503_ = 13.78, *p* < 0.001; Figure 2). Additionally, the number of entrances per trap did not significantly impact bee captures among the same size of entrances (T5mm-20–T5mm-10 p_adj_ = 0.96; T4mm-20–T4mm-10 p_adj_ = 0.64).

A total of 33 ladybirds (18 *H. axyridis* and 15 *C. septempunctata*) were captured. Traps with 6 mm entrances captured significantly more ladybirds compared to traps with 4 mm entrances (T6mm-20–T4mm-10 p_adj_ < 0.01; T6mm-20–T4mm-20 p_adj_ < 0.01), but not with traps with 5 mm entrances (T6mm-20–T5mm-10 p_adj_ = 0.14; T6mm-20–T5mm-20 p_adj_ = 0.33; Figure 2). There was no significant difference in ladybird captures between traps with 4 mm entrances and those with 5 mm entrances (ANOVA F_4,499_ = 4.31, *p* < 0.01; Figure 2). A post hoc Tukey test showed that the number of entrances per trap did not significantly impact ladybird captures (T5mm-20–T5mm-10 p_adj_ = 0.99; T4mm-20–T4mm-10 p_adj_ = 1.00).

A total of 240 hoverflies (210 *Toxomerus* sp. (Macquart), 7 *Allograpta* sp. (Osten Sacken), 16 *Syrrita* sp. (Le Peletier & Serville), 4 *Rhingia* sp. (Scopoli), 1 *Eristalis* sp. (Latreille), and 2 *Eupeodes americanus* (Wiedemann) were captured. There were no significant differences in hoverfly captures among the different treatments (ANOVA F_4,501_ = 0.96, *p* = 0.43; Figure 2).

### 3.3. Selection of the Optimal Lure

**Figure 3:**
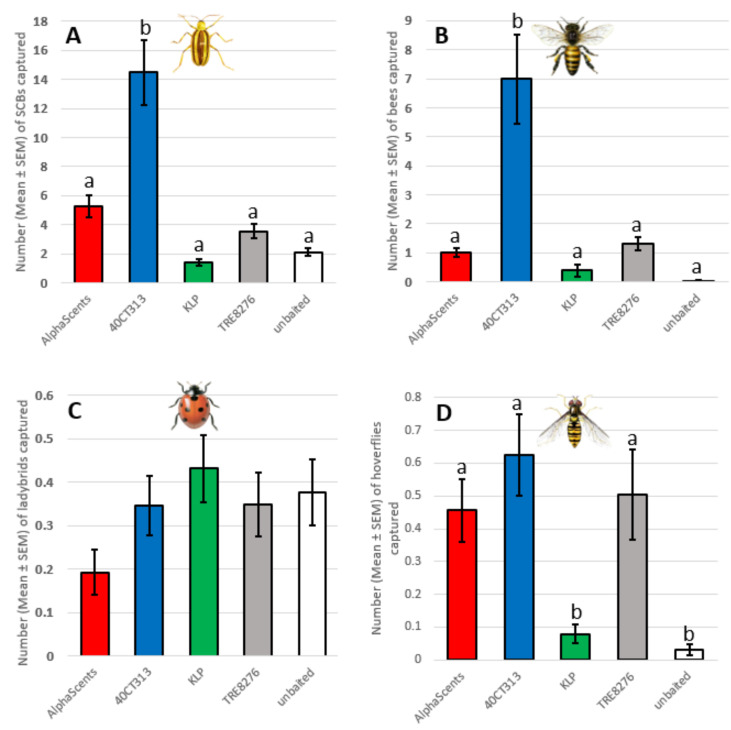
Number of SCBs, ladybirds, bees and hoverflies captured in traps baited with different lures (ANOVA, (**A**) SCB—F_4,505_ = 22.02, *p* < 0.001; (**B**) bees—F_4,505_ = 15.55, *p* < 0.001; (**C**) ladybirds—F_4,505_ = 1.55, *p* = 0.19; (**D**) hoverflies—F_4,505_ = 7.49, *p* < 0.001). Different letters above bars indicate significant differences according to the Tukey HSD test (*p* < 0.05).

A total of 2734 SCBs were captured in this experiment. Traps baited with 40CT313 attractants captured significantly more SCBs than all other treatments (AlphaScents, KLP, TRE8276 and unbaited) (ANOVA F_4,505_ = 22.02, *p* < 0.001; Figure 3).

A total of 992 bees (553 *A. mellifera*, 53 *Bombus* sp. and 386 *P. pruinosa*) were captured. Traps baited with 40CT313 attractants captured significantly more bees than other treatments (ANOVA F_4,505_ = 15.55, *p* < 0.001; Figure 3). All the traps used in this this experiment had two series of ten holes of 6 mm in diameter per side, i.e., the largest opening tested of the first experiment.

A total of 173 ladybirds (18 *P. quatuordecimpunctata*, 28 *C. maculata*, 99 *H. axyridis*, 26 *C. septempunctata,* and 2 *Hippodamia* sp.) were captured. There were no significant differences in captures between treatments (ANOVA F_4,505_ = 1.55, *p* = 0.19; Figure 3).

A total of 173 hoverflies were captured (137 *Toxomerus* sp., 12 *Allograpta* sp., 12 *Syrrita* sp., 1 *Rhingia* sp., 5 *Eristalis* sp., and 6 *E. americanus*). Traps baited with AlphaScents, 40CT313 and TRE8276 captured significantly more hoverflies than traps baited with KLP or unbaited traps (ANOVA F_4,505_ = 7.49, *p* < 0.001; Figure 3).

## 4. Discussion

The main objective of this study was to optimize a SCB trapping method for use in organic cucurbit fields. The optimal trapping method maximizes SCB captures while limiting non-target insect captures. SCB are primary insect pests of cucurbits crops in North America, and alternative control methods are needed due to the increasing limitations of pesticides [39]. The trapping method’s efficacy was established previously in US conventional cucurbit fields by Piñero and Lee (2016) [19]. However, in contrary to this study, many non-target insects were captured in the organic fields. Our results showed that baited traps attract significantly more SCBs, bees, and hoverflies than unbaited traps in organic cucurbit crops. Traps with ten 4 mm in diameter openings per side baited with the 40CT313 attractant optimized SCB captures, while reducing non-target insect captures.

Overall, the 36 traps captured over 1900 SCBs over a nine-week period (3 July to 28 August 2018) during the first experiment, showing that this trapping method can capture SCBs in organic cucurbit crops. Comparatively, Piñero and Lee (2016) had reported 15 traps capturing over 3200 SCBs over the course of 6 weeks (29 April to 14 June 2016), with ratios of SCBs in traps versus SCBs on plants ranging between 7:1 and 23:1. The average amount of SCB per plant had also been greatly suppressed but did not always reduce SCB numbers under the economic threshold [19]. Such ratios and threshold were not examined in our study since the main objective was to determine if baited traps captured more SCBs than unbaited traps, and which trap type and lure could maximize SCB captures while minimizing non-species captures. While our results might be less impressive than Piñero and Lee’s, it is important to consider that half the traps used in our study were unbaited, the other half being baited with Trécé TRE8276 lure, whereas Piñero and Lee had used AgBio SCB lure in all their traps. AgBio SCB lure and its equivalent, 40CT313, were not available in Canada in 2018 when the first experiment took place, which could contribute to SCB captures being lower in our first experiment. Furthermore, our experiment took place in a different geographical context, as well as in organic cucurbit productions, whereas Piñero and Lee tested in Missourian conventional cucurbit fields. Finally, stun pills, which are permitted for conventional but not organic use, were used to stun SCBs in Piñero and Lee’s experiment, whereas we traps were filled with unscented soapy water to drown the SCBs in our experiment, potentially giving more opportunities for SCBs to escape the traps when entering.

Results from this experiment also indicated that traps baited with semiochemical-based attractants captured 1.70 times more SCBs than unbaited traps. Baited traps also captured 1.20 times more ladybirds, 3.57 times more bees, and 2.16 times more hoverflies than unbaited traps. Insects use both visual and olfactory cues when foraging, making a trapping method using both stimuli more efficient in capturing pest insects such as SCBs, but also non-target insects like pollinators [35,40]. Both conventional and organic cucurbit productions depend on pollinators to produce fruit, as cucurbits have entomophilic pollination [20]. Thus, the second experiments of this research aimed to reduce these unwanted captures.

The trap type experiment demonstrated that traps perforated with ten 4 mm in diameter openings per side efficiently captured SCB while minimizing non-target species captures. Piñero & Lee (2016) have previously established that yellow jugs with thirty 6 mm in diameter openings made with a paper-hole punch were optimal for SCB trapping in comparison to 10 cm long and 3 mm wide slots made with a Dremel tool or similar slots made with a knife to maximize SCB captures and exclude honeybees [19]. Our experiment showed that traps with 6 mm in diameter openings should be avoided, since they trapped significantly more pollinators and natural enemies, whereas the 4 mm opening traps significantly limited these non-target insects captures. The tool used to make the openings by Piñero and Lee was different, since they used a paper-hole punch whereas a soldering iron was used for this study.

The trap type experiment was the only experiment having blocks as a significant factor for capturing SCBs. This significance could be caused by the disposition of the blocks, as some blocks were closer to paved or dirt roads both farms. Hibernation sites of SCBs are still unclear; however, they have been reported to emerge from leaf litter and dense weeds to feed on alternate host plants until cucurbits are available [3,4]. Roads are barren and provide no refuge nor food for SCBs, which could explain why blocks were a significant factor in this experiment. The type of cucurbit planted within the fields also varied, as both farms mixed different varieties of cucurbits in their crops which could also explain the difference between blocks.

The attractant experiment showed that the 40CT313 lure attracted significantly more SCBs than other lures. The attractant 40CT313 is an equivalent of the AgBio 5-compound lure P313-B5, which was previously documented as being the most attractive lure to SCBs by Piñero (2018) [35]. This lure also attracted more bees than other attractants and was more attractive for hoverflies, which was not previously documented. These unwanted captures can be drastically reduced by using the optimal trap from the trap type experiment.

Additionally, over 1700 western corn rootworm beetles, *Diabrotica virgifera virgifera* (Coleoptera: Chrysomelidae), were captured in the traps containing 40CT313. The western corn rootworm beetles are a problematic insect in corn production, and this unexpected finding could provide insight to finding alternative ways to control this pest.

The purpose of this study was to establish the best trap type and lure for mass trapping SCBs in organic cucurbit farms. Traps with ten 4 mm holes per side with the 40CT313 attractant are the most effective at capturing SCBs while reducing non-target species captures in organic cucurbit farms. While these results are promising, further studies are necessary to establish whether the mass trapping technique of SCBs will effectively maintain SCB populations under the economic thresholds in organic cucurbit fields or not.

## Data Availability

The data presented in this study are available on request from the corresponding author. The data are not publicly available due to privacy restrictions.
